# 

**Published:** 1951-01

**Authors:** 


### The Bristol Medico-Chirurgical Journal

A JOURNAL OF THE MEDICAL SCIENCES FOR THE

### West of England and South Wales

PUBLISHED UNDER THE AUSPICES OF

*THE BRISTOL MEDICO-CHIRURGICAL SOCIETY*

EDITOR:

E. WATSON-WILLIAMS, *M.C.*, M.D., Ch.M., F.R.C.S.

WITH WHOM ARE ASSOCIATED

G. E. F. SUTTON, *M.C.*, M.D., F.R.C.P.

J. H. MIDDLEMISS, M.D., D.M.R.D., F.F.R.

N. S. CRAIG, *M.C.*, M.D., *Assistant Editor*

---

"Scire est nescire, nisi id me

Scire alius sciret."

---

1951 *VOL. LXVIII*

BRISTOL: J. W. ARROWSMITH LTD.

WI
BR323

### 1951 Contents of Vol. LXVIII

*page*

The Presidential Address: Some Surgical Milestones. By W. A. Jackman, *T.D.*, F.R.C.S. ... ... ... ... ... ... ... ... 1

The Thirty-Ninth Long Fox Memorial Lecture: Some Food Infection Problems. By Sir William Savage, M.D., B.Sc. ... ... ... 9

Streptomycin. By Beryl Corner, M.D., M.R.C.P., and Seymour Mason, M.B., B.S., D.C.H. ... ... ... ... ... ... 16

Keratosis Pharyngis. By E. Watson-Williams, *M.C.*, M.D., Ch.M. ... 23

Geriatrics. I: The Medical Problem of To-day. By C. T. Andrews, M.D., F.R.C.P. II: Modern Developments. By T. S. Wilson, M.D. III: The Local Problem. By William Hughes, M.D., M.R.C.P. 35

Liver Function Tests. By G. Kemp McGowan, B.A., B.M., B.Ch. ... 46

Surgical Neurology. By George L. Alexander, F.R.C.S. ... ... 55

The Atomic Bomb. I: The Release of Atomic Energy and the Pathological Effects of Radiation. By R. C. Tudway, M.B., B.S., B.Sc., D.M.R. II: The Effects of the Explosion. By John H. Challenger, M.B., Ch.B., . ... ... ... ... ... ... ... ... 71

Reiter's Syndrome. By A. E. W. McLachlan, M.B., D.P.H., F.R.S.E. 87

Backache, Sciatica and Ruptured Discs. By Kenneth Pridie, F.R.C.S. 95

Animal Disease in Relation to Man. By A. M. G. Campbell, D.M., F.R.C.P. ... ... ... ... ... ... ... ... ... 105

The Journal: A Swan-Song. By E. Watson-Williams, *M.C.*, M.D. Ch.M. ... ... ... ... ... ... ... ... ... 116

The Diagnosis and Treatment of Congenital Heart Disease. By R. Milnes Walker, M.S., F.R.C.S., and John Apley, M.D., M.R.C.P. ... 124

Editorial Notes ... ... ... ... ... ... ... 25, 98, 139

Obituary ... ... ... ... ... ... ... ... ... 58

Meetings of Societies ... ... ... ... ... 27, 60, 100, 139

Local Medical Notes ... ... ... ... ... 32, 62, 102, 144

**In Memoriam**

JAMES FRANCIS BLACKETT

M.D., M.A., B.S., D.P.H.

### Local Medical Notes

The Thirty-Ninth Long Fox Memorial Lecture was delivered in the University on Tuesday, October 17th, by Sir William G. Savage (p. 9). The chair was taken by Professor T. Hewer: six members of the Appointing Board were present, and thirty-three others—*rari in gurgite vasto.* The lecturer, as others have done, found difficulty in dealing with somewhat technical matter with a non-medical audience: such terms as "vi-agglutinin" and "enterotoxin" are not likely to be understood unless carefully explained—probably not even then!

BRISTOL MATERNITY HOSPITAL

On Thursday, October 19th, Mrs. Winston Churchill, in a black velvet beret and a carefully impromptu speech, declared open the new Bristol Maternity Hospital at the corner of Durdham Downs, formerly the Queen Victoria Convalescent Home. Mr. H. L. Shepherd gave a most felicitous description of his encounter with that very rare bird, a "gold-crested W.R.N". The buildings were inspected and admired, the "gadget" that attracted most attention being the microphone fitted beside each bed to summon assistance.

SIR HUMPHRY DAVY

In memory of Sir Humphry Davy, described as "The father of Anaesthesia", a Tablet was unveiled on October 27th on the house at 6 Dowry Square, Hotwells; where he worked at the end of the eighteenth century as assistant to Dr. Beddoes, and discovered the anaesthetic properties of nitrous oxide. Dr. R. Woolmer was present, representing the United Bristol Hospitals. Representatives of the University, the Faculty of Medicine, the non-teaching hospitals, the local medical associations, societies and journals were not present: perhaps because they had not been invited. After all, the organisers probably did not suspect that any of these would be interested (cf. *Jl.* 1923, XL, 113).

### List of Subscribers

It is proposed to publish the list of subscribers every third year, instead of annually. Unfortunately the list due this year is not yet ready, but will appear in the next issue.

### Appointments

Mr. A. W. Adams, M.S., F.R.C.S.—Hunterian Professor, Royal College of Surgeons.

Professor J. M. Yoffey, M.D., F.R.C.S.—Arris and Gale Lecturer, Royal College of Surgeons.

Dr. Macdonald Critchley, F.R.C.P.—Visiting Professor in Neurology, University of Istanbul.

Dr. N. G. B. McLetchie.—Professor of Pathology, Dalhousie University, Halifax, Nova Scotia.

Mr. E. K. Tratman.—Professor of Dental Surgery, University College Hospital, London.

Dr. G. A. Smart, M.R.C.P.—Reader in Medicine at Durham University.

Professor R. J. Brocklehurst, M.A., D.M.—President, Physiology Section, British Association.

Dr. J. N. P. Davies.—Head of Department of Pathology at the Medical School, Kampala, Uganda.

Dr. Enid Addenbrooke, M.R.C.P., D.C.H.—Consultant Paediatrician, N. Gloucester Area.

C. H. Bartlett, F.R.C.S.—Consultant Surgeon, Bristol.

P. H. Beamish, D.M.R.D.—Consultant in Radiodiagnosis, Newcastle-on-Tyne.

D. R. F. Bertram, *O.B.E.*, M.B., D.M.R.—Consultant Radiologist, S. Somerset.

T. A. Brand, M.D.—Paediatrician, Newport and Monmouthshire Hospitals.

Roma Chamberlain, M.B., D.C.H.—Assistant M.O.H., Reading.

F. L. Dyson, M.D., M.R.C.P.—Consultant Physician, Mid-Glamorgan Hospital Group.

H. L. Ellis, M.D., M.R.C.P.—Consultant Paediatrician, Radcliffe Infirmary, Oxford.

G. Garmany, M.R.C.P., D.P.M.—Consultant Psychiatrist, Belmont Hospital, Surrey.

G. M. Gibb, M.B., D.P.M.; J. B. Phillips, M.D., D.P.M.—Psychiatrists, Bristol Mental Hospital.

H. W. Hall, F.R.C.S.—Consultant Orthopaedic Surgeon, Bath Area.

D. Jackson, M.B., D.P.M.—County Psychiatrist, Cornwall.

A. P. C. D. Thomson, D.M.R.—Consultant Radiologist, Bath Area.

G. H. Valentine, M.R.C.P.—Consultant Paediatrician, Peterborough Area.

J. C. Valentine, M.B.—Consultant Pathologist, Bedford General Hospital.

H. F. West, M.D., M.R.C.P.—Consulting Physician in Rheumatic Diseases, United Sheffield Hospitals.

### Scholarships and Prizes

*George Fawn Prize.*—H. T. Jones.

*David Rayner Prize.*—W. A. W. Dutton.

*Thomas Francis Edgeworth and Francis Henry Edgeworth Prize.*—G. A. J. Aycliffe.

*Bristol Royal Hospital Board's Gold Medal.*—J. D. Sheward.

*Bristol Royal Hospital Board's Silver Medal.*—D. M. Gambier.

### Degrees and Diplomas

**University of Cambridge.**—M.D.: A. Miller.

**University of London.**—M.D.: A. G. Freeman.

**Royal College of Surgeons.**—F.R.C.S.: J. C. Ballantyne. F.D.S.: W. E. Snawdon.

**Royal Colleges of Physicians and Surgeons (Conjoint Board).**—M.R.C.S., L.R.C.P.—Ginette M. Tebbutt.

